# NOTES FROM THE FIELD: TAPPING INTO RESILIENCE THROUGH REFLECTIVE WRITING

**DOI:** 10.1093/geroni/igz038.850

**Published:** 2019-11-08

**Authors:** Holly M Holmes, Thomas R Cole

**Affiliations:** UT Houston McGovern Medical School, Houston, Texas, United States

## Abstract

Reflective writing is a powerful tool that can help healthcare providers address burnout and access inner strength. We describe the formation and functioning of a writing group for palliative care and geriatrics physicians at McGovern Medical School. The group has served several functions, including the promotion professional and personal growth. Using reflective writing and prompts, our group has explored issues of compassion, caregiving, grief, and loss. Group writing has provided a safe space for processing and letting go of professional and personal stressors related to caring for patients and to demands in our daily lives. The positive impact of the writing group has extended to caregiving and to other writing, including technical writing. This symposium is designed for healthcare professionals in all disciplines who are interested in exploring the use of reflective writing in regular practice.

